# Is Alteration of Tuning Property in Cervical Vestibular-Evoked Myogenic Potential Specific for Ménière’s Disease?

**DOI:** 10.3389/fneur.2017.00193

**Published:** 2017-05-08

**Authors:** Toshihisa Murofushi, Masahito Tsubota, Ryota Suizu, Eriko Yoshimura

**Affiliations:** ^1^Department of Otolaryngology, Teikyo University School of Medicine Mizonokuchi Hospital, Kawasaki, Japan; ^2^Yoshimura ENT Clinic, Fujisawa, Japan

**Keywords:** endolymphatic hydrops, Ménière’s disease, tuning, saccule, otolith organ, vertigo

## Abstract

**Objective:**

The aim of this study is to show sensitivity and specificity of cervical vestibular-evoked myogenic potential (cVEMP) tuning property test to Ménière’s disease (MD) in comparison with healthy controls (HC) and patients with other vestibular diseases.

**Subjects:**

Totally 92 subjects (50 women and 42 men, 20–77 years of age) were enrolled in this study. Subjects were composed of 38 definite unilateral MD patients, 11 unilateral benign paroxysmal positional vertigo patients, 14 vestibular migraine patients, 19 unilateral vestibular neuritis patients, and 10 HC.

**Methods:**

The subjects underwent cVEMP testing to 500 and 1,000 Hz short tone bursts (125 dBSPL). The corrected amplitudes of the first biphasic responses (p13–n23) (cVEMP) were measured. Then, a tuning property index (the 500–1,000 Hz cVEMP slope) was calculated.

**Results:**

The area of under the ROC curve (AUC) was 0.75 in comparison with other vestibular disease patients, while AUC was 0.77 in comparison with other vestibular disease patients plus HC. The best cutoff point of the 500–1,000 Hz cVEMP slope was −19.9. Sensitivity of the tuning property test to MD was 0.74, while specificity was 0.76 to other vestibular disease patients.

**Conclusion:**

The tuning property test of cVEMP is useful as a screening test of MD.

## Introduction

Ménière’s disease (MD) is one of representative peripheral vestibular diseases. MD is characterized by episodic vertigo attacks, fluctuating hearing loss, tinnitus, and aural fullness. Although its exact pathophysiology remains unclear, MD has been recognized as an idiopathic syndrome of endolymphatic hydrops (EH) (1). A method for detecting EH using magnetic resonance (MR) imaging has been developed (2). MR imaging requires high costs and is currently used in a research setting (3). Therefore, the physiological confirmation of EH is still useful for diagnosis of EH in MD (4). For detection of EH in the cochlea, electrocochleography (5) and glycerol test using improvement of pure-tone hearing as an index (6) have been used, while for detection of EH in the semicircular canal, furosemide test has been used (7). However, the detection methods of EH in the otolith organs including the saccule have been rarely reported.

Recently, Murofushi et al. reported methods for detection of EH in the saccule, which has been reported to exhibit EH most frequently among the structures of the vestibular labyrinth (8, 9). The one is the glycerol cervical vestibular-evoked myogenic potential (cVEMP) test, and the other is the tuning property test of cVEMP (4). Traditionally, glycerol has been used for the test of EH in the cochlea because glycerol temporally reduces EH in the inner ear (6). Then, improvement of pure-tone hearing after glycerol administration was regarded as a sign suggestive of EH in the cochlea. Recently, glycerol administration has been applied to cVEMP test, a test of functions of the saccule, to detect EH in the saccule (4). As glycerol cVEMP test, improvement of cVEMP responses was assessed after intravenous infusion of 10% glycerol (500 ml, 2 h) (4). The positivity rate (PR) of glycerol cVEMP test was 60% (12/20) in definite MD patients when significant improvement of cVEMP amplitude (p13–n23) was adopted as an index.

The tuning property test is another test for detection of EH in the saccule. As Rauch et al. reported (10), MD patients tend to show 1,000-Hz dominant cVEMP responses in comparison with cVEMP responses to 500 Hz, while healthy subjects show 500-Hz dominant cVEMP responses. Murofushi et al. calculated 500–1,000 Hz cVEMP slope to quantify this frequency preference (see Methods in this article) (11). According to Murofushi et al. (4), PR of tuning property test in definite MD ears was 69% (11/16) when totally cVEMP-absent patients were excluded. In that study, the cutoff point was set using mean − 2SD of the healthy subjects. Furthermore, results of the tuning property test almost corresponded to those of the glycerol cVEMP test except for patients with no cVEMP response. The tuning property test, which only requires recording cVEMP to 500 and 1,000 Hz short tone bursts (STBs), seems to be practically useful as an easy way of EH detection in the saccule because physiological EH detection test has never been reported except for glycerol cVEMP test, which is much more time consuming than tuning property test. Although PR of tuning property test (=69% in the previous study) (4) sounds low, it should be noted that EH in the vestibule was detected only in 70% of definite MD patients even though gadolinium-enhanced MRI was applied (12).

Although the tuning property test seems to be able to differentiate MD patients from healthy controls (HC) (11), its specificity in comparison with other types of vestibular diseases such as benign paroxysmal positional vertigo (BPPV) and vestibular migraine (VM) is still unclear. Herein, we studied sensitivity and specificity of cVEMP tuning property test to MD in comparison with patients with other vestibular diseases.

## Materials and Methods

### Subjects

Totally 92 subjects (50 women and 42 men, 20–77 years of age) were enrolled in this study. Subjects were composed of 38 definite unilateral MD patients (AAO-HNS 1995) (1), 11 unilateral BPPV patients (13), 14 VM patients (14), 19 unilateral vestibular neuritis (VN) patients (15), and 10 healthy volunteers (Table 1). Healthy volunteers did not have medial history of otological, audiological, or vestibular disorders. Although we recruited MD patients according to AAO-HNS 1995 criteria (1), all the definite MD patients in this study fulfilled diagnostic criteria published by Barany Society in 2015 (16) as well. Although BPPV patients were recruited by traditional diagnostic criteria, all the BPPV patients in this study fulfilled diagnostic criteria published by Barany Society in 2015 (13).

**Table 1:** **Age and gender in each group**.

	*N* (bodies)	Men	Women	Mean age
Ménière’s disease	38	17	21	53.6
Benign paroxysmal positional vertigo	11	3	8	47.8
Vestibular migraine	14	4	10	40.4
Vestibular neuritis	19	12	7	54.2
Healthy controls	10	6	4	35.2

Total	92	42	50	49.0

### Methods

The cVEMP recording was conducted as follows (4, 15). The recording was basically performed according to the international guidelines for cVEMP recording (17). The Neuropack system (Nihon Kohden Co. Ltd., Japan) was used to record the cVEMP. Electrodes were placed on the upper half of each sternocleidomastoid muscle (SCM), with a reference electrode placed on the lateral end of the upper sternum and a ground electrode placed on the nasion. During the recording procedure, the subjects were asked to lie in the supine position and raise their heads to contract the SCM. As acoustic stimuli, air-conducted 500 and 1,000 Hz STBs (125 dBSPL, rise/fall time = 1 ms, plateau time = 2 ms) were presented through headphones (type DR-531, Elega Acoustic Co. Ltd., Japan) at a stimulation rate of 5 Hz. The signals were amplified and bandpass filtered (20–2,000 Hz), and 100 responses were averaged. The time window for the recording ran from −20 to 80 ms. To confirm the reproducibility of the results, two runs were performed for each ear. The first biphasic responses (p13–n23) produced by the SCM ipsilateral to the stimulated ear were assessed. To eliminate the effects of variations in muscle activity, the mean background amplitude was calculated from the mean rectified background activity during the 20-ms prestimulus period. The corrected (normalized) cVEMP amplitude (dimensionless) was calculated as follows and employed for comparisons.

$$\begin{array}{l} \text{Corrected }(\text{normalized})\text{ amplitude}=(\text{raw amplitude})/(\text{mean rectified background}\\ \text{ amplitude during 20 ms prestimulus period)}. \end{array}$$

In the tuning property test (4), the corrected (normalized) amplitude of the p13–n23 produced in response to the 500 Hz STB was compared with the corrected (normalized) amplitude of the p13–n23 produced in response to the 1,000-Hz STB. The 500–1,000 Hz cVEMP slope was calculated as a tuning property index (a measure of frequency preference) as follows:

$$\begin{array}{l} 500-1,000\text{ Hz cVEMP slope = 100}\times (\text{CA}500-\text{CA}1000)/(\text{CA}500+\text{CA}1000). \end{array}$$

where CA500 (1,000) = corrected (normalized) amplitude of the p13–n23 produced in response to the 500 (1,000) Hz STB. When both the CA500 and CA1000 were 0, the 500–1,000 Hz cVEMP slope was regarded as “uninformative.” Here, “uninformative” implies that no information concerning tuning property was obtained because no response was observed to either frequency.

## Results

**Figure 1:**
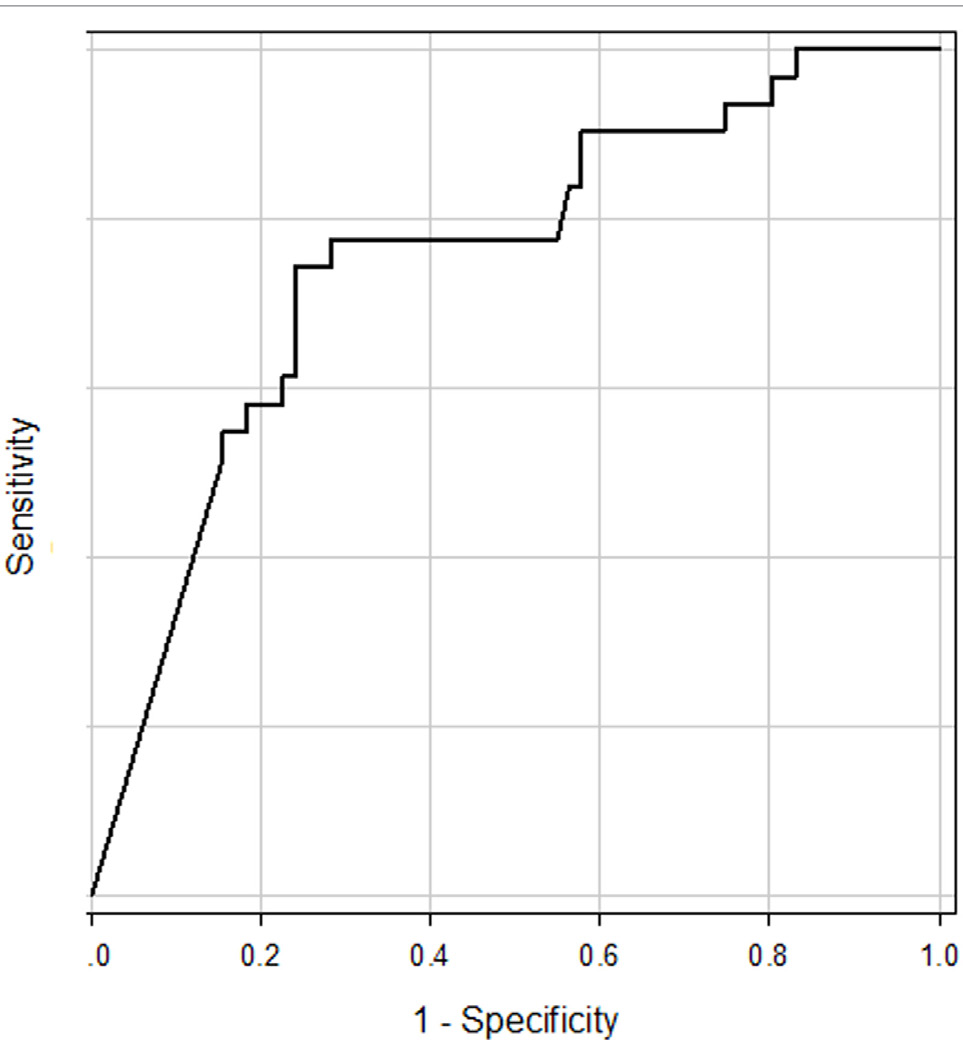
**ROC curve for prediction of Ménière’s disease patients from other vestibular disease patients**. The area of under the ROC curve was 0.75.

First, ROC curves (receiver operating characteristic curves) were constructed for assessment of accuracy of the tuning property test and confirmation of the best cutoff point for differentiation of MD (Figure 1) (18). In comparison with other vestibular disease patients, the area of under the ROC curve (AUC) was 0.75 (95% CI 0.64–0.85). As a reference, we also constructed ROC curve in comparison with other vestibular disease patients plus HC. AUC was 0.77 (95% CI 0.68–0.88). The best cutoff points suggested by ROC curves were −19.9 to both groups (using the Youden index) (18).

**Figure 2:**
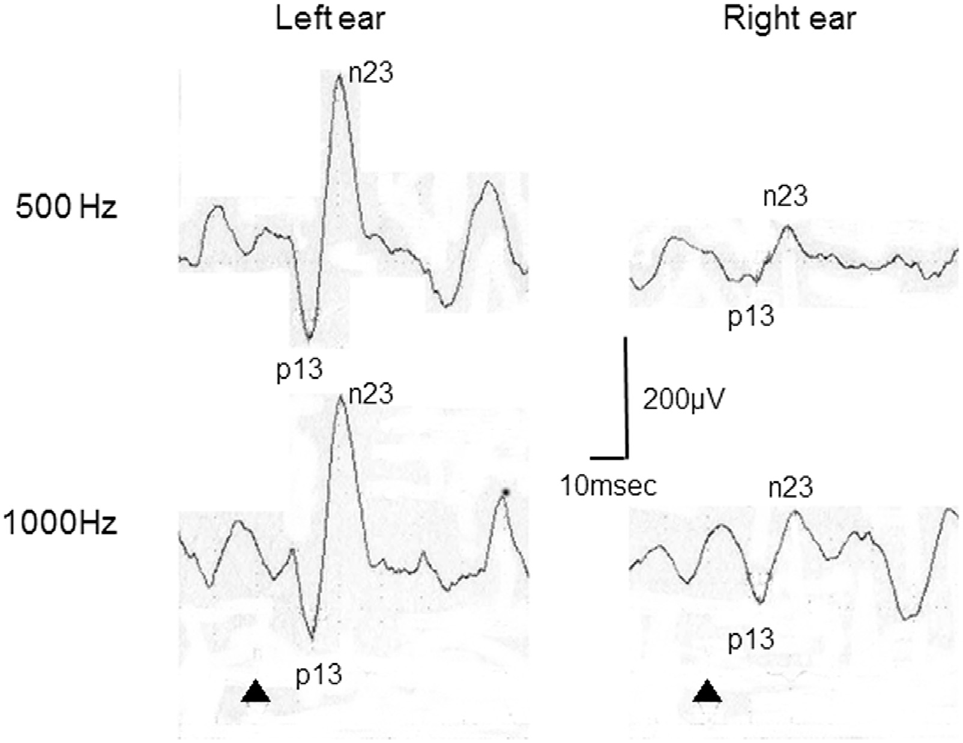
**Representative cervical vestibular-evoked myogenic potential (cVEMP) responses tuning property test positive**. This patient was a 22-year-old woman with right Ménière’s disease. In this case, 500–1,000 Hz cVEMP slopes were −29.8 in the right and 0 in the left.

**Figure 3:**
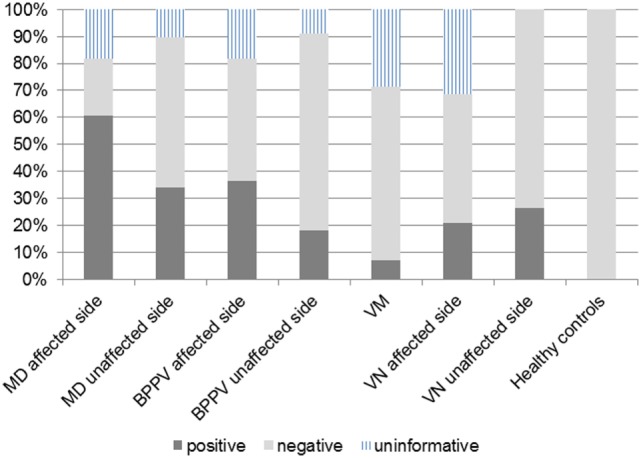
**Positivity rate (PR) in each group**. The cutoff point of 500–1,000 Hz cervical vestibular-evoked myogenic potential slope was −19.9. Definite Ménière’s disease (MD)-affected ears showed the highest PR.

According to results of ROC curve study, the cutoff point was set as −19.9. When the 500–1,000 Hz cVEMP slope was ≤−19.9, the tuning property test was regarded as positive, suggestive of saccular EH (Figure 2). The results of the tuning property test are summarized in Table 2 and Figure 3. PR (=sensitivity of the test) was the highest in the MD-affected ears (PR = 60 in inclusion of the uninformative as negative and 74 in exclusion of the uninformative) (χ^2^ test, *p* < 0.001).

**Table 2:** **Positivity rate in each group**.

	*N* (ears)	Positive (*P*)	Negative (*N*)	Uninformative (*U*)	PR [100 × *P*/(*P* + *N* + *U*)]	PR^a^[100 × *P*/(*P* + *N*)]
MD-affected side	38	23	8	7	60.5	74.2
MD-unaffected side	38	13	21	4	34.2	38.2
BPPV-affected side	11	4	5	2	36.3	44.4
BPPV-unaffected side	11	2	8	1	18.1	20.0
VM	28	2	18	8	7.1	10.0
VN-affected side	19	4	9	6	21.0	30.7
VN-unaffected side	19	5	14	0	26.3	26.3
HC	20	0	20	0	0	0

*PR, positivity rate; MD, Meniere’s disease; BPPV, benign paroxysmal positional vertigo; VM, vestibular migraine; VN, vestibular neuritis; HC, healthy controls*.

*^a^PR in exclusion of uninformative ears*.

In comparison between the MD-affected ears and the MD-unaffected ears, PR of the MD-affected ears was significantly higher than the MD-unaffected ears (χ^2^ test, *p* < 0.01).

Specificities were assessed to results of ears of other vestibular disease patients and those of other vestibular disease patients plus HC, respectively (Tables 3 and 4). Specificities were 0.76 for other vestibular disease patients and 0.81 for other vestibular disease patients plus HC.

**Table 3:** **Sensitivity and specificity to other vestibular disease patients**.

	Positive	Negative	Total
Ménière’s disease-affected side	23	8	31
Other vestibular diseases	17	54	71

Total	40	62	102

**Table 4:** **Sensitivity and specificity to other vestibular disease patients plus healthy controls (HC)**.

	Positive	Negative	Total
Ménière’s disease-affected side	23	8	31
Other vestibular diseases plus HC	17	74	91

Total	40	82	122

Mean ages among the groups showed significant differences (*p* < 0.001, one-way ANOVA). Pairwise multiple comparison procedures (Holm–Sidak method) revealed that significant differences (*p* < 0.05) were observed between MD and VM, between MD and HC, between VN and HC, and between VN and VM.

## Discussion

This study revealed that the tuning property test of cVEMP has moderate accuracy for differentiation of MD from patients with other vestibular diseases (and HC). To study specificity of the tuning property test not only for healthy subjects but also for patients with heterogeneous vestibular pathologies, we compared results of definite MD patients with those of such heterogeneous population, because, at clinics, we perform differential diagnoses not between MD and the healthy but between MD and other vestibular pathologies.

Furthermore, the current study suggested that the best cutoff point of 500–1,000 Hz cVEMP slope for differentiation of MD should be −19.9. Murofushi et al. have used −19.6 as a cutoff point because it corresponded to mean − 2SD of HC (11). As a consequence, the cutoff point suggested by ROC in this study was very close to the value used in the previous study (−19.6) (4, 11).

Rauch et al. reported that patients with MD exhibit a different cVEMP tuning pattern from healthy subjects (10). In their study, the healthy subjects showed best cVEMP responses around 500 Hz, while the MD patients exhibited greater responses at 1,000 Hz than at 500 Hz on the affected side. Node et al. found that the same frequency preference observed in MD patients was normalized by furosemide loading (19). These studies suggested that an altered cVEMP tuning pattern could be a marker of saccular EH. Murofushi et al. proposed that the 500–1,000 Hz cVEMP slope could be used as an index of saccular EH (4, 11, 20, 21). Kim-Lee et al. reported the similar tendency in MD patients (22). Hereafter, this tuning pattern test is referred to as the “tuning property test” (4). Similar alteration of tuning property was also reported in ocular VEMP (oVEMP) (23).

Although previous studies have suggested difference of cVEMP tuning property between MD patients and HC, one of clinically important points is differentiation of MD from other vestibular diseases. This study showed that the tuning property test has moderate accuracy for differentiation of MD patients from patients with other vestibular diseases (AUC = 0.75, specificity = 0.76 for −19.9 as the cutoff point).

Differentiation between MD and VM is sometimes difficult. The tuning property test could be a useful diagnostic tool for this differentiation. According to Nakada et al. (24), two of seven patients with VM showed vestibular EH in gadolinium-enhanced MRI. Their findings imply that specificity of gadolinium-enhanced MRI of MD in comparison with VM was 0.71. VM patients with positive results in the tuning property test or gadolinium-enhanced MRI might be diagnosed as MD/VM overlapping syndrome in the future (16).

Another point of interest of this study was a moderate PR of BPPV patients in the tuning property test. It was reported that patients with BPPV could have abnormal cVEMP and oVEMP probably due to some pathological changes in the otolith organs (25, 26). It is well known that MD patients could present BPPV-like vertigo attacks (27, 28). According to a report of Hughes and Proctor, 45 (29.8%) of 151 patients had an associated diagnosis of MD in a retrospective review of a large population of BPPV patients (29). Therefore, BPPV-like vertigo attacks might be the first presentation of vertigo due to EH, followed by the development of MD. Concerning association of MD with BPPV, it was also proposed that both diseases might be caused by detached otoconia (30).

While the tuning property test is an easy way for detection of saccular EH, it has limitations. The tuning property test does not provide any information concerning EH when the subjects showed absence of cVEMP to both 500 and 1,000 Hz STB. In these cases, the glycerol cVEMP test is required (4). Bilateral absence of cVEMP in the elderly could be due to aging (31).

Secondary, the mean ages among some groups showed significant differences. These age differences might affect results. However, PR of the MD-affected ears was significantly higher than the MD-unaffected ears. It is unlikely that age differences might be a major factor of results. Third, the number of subjects in each subgroup of other vestibular diseases was limited. As a next step, a larger-sized study will be required.

## Ethics Statement

Informed consent was obtained from each subject, and ethical approval was received from the ethics committee of Teikyo University (TR14-098, 15 October 2014).

## Author Contributions

All authors contributed extensively to the work presented in this paper. All authors collected data. TM wrote the manuscript. MT, RS, and EY reviewed and edited the manuscript.

## Conflict of Interest Statement

The authors declare that the research was conducted in the absence of any commercial or financial relationships that could be construed as a potential conflict of interest.
